# Early oral switch therapy in low-risk *Staphylococcus aureus* bloodstream infection (SABATO): study protocol for a randomized controlled trial

**DOI:** 10.1186/s13063-015-0973-x

**Published:** 2015-10-09

**Authors:** Achim J. Kaasch, Gerd Fätkenheuer, Reinhild Prinz-Langenohl, Ursula Paulus, Martin Hellmich, Verena Weiß, Norma Jung, Siegbert Rieg, Winfried V. Kern, Harald Seifert, for the SABATO trial group (with linked authorship to the individuals in the Acknowledgements section)

**Affiliations:** Institute for Medical Microbiology, Immunology and Hygiene, University of Cologne, Goldenfelsstr. 19-21, D-50935 Köln, Germany; Department I of Internal Medicine, University Hospital of Cologne, 50924 Cologne, Germany; Clinical Trial Center Cologne, University of Cologne, 50924 Cologne, Germany; Institute of Medical Statistics, Informatics and Epidemiology, University of Cologne, Kerpener Straße 62, 50924 Cologne, Germany; Department of Medicine, Center for Infectious Diseases and Travel Medicine, University Medical Center Freiburg, Hugstetter Straße 55, 79106 Freiburg, Germany; German Centre for Infection Research (DZIF), partner site Bonn-Cologne, Germany

**Keywords:** Oral switch therapy, Intravenous, Antimicrobial, Bloodstream infection, Bacteremia, *Staphylococcus aureus*, Randomized controlled trial, Pragmatic trial

## Abstract

**Background:**

Current guidelines recommend that patients with *Staphylococcus aureus* bloodstream infection (SAB) are treated with long courses of intravenous antimicrobial therapy. This serves to avoid SAB-related complications such as relapses, local extension and distant metastatic foci. However, in certain clinical scenarios, the incidence of SAB-related complications is low. Patients with a low-risk for complications may thus benefit from an early switch to oral medication through earlier discharge and fewer complications of intravenous therapy.

The major objective for the SABATO trial is to demonstrate that in patients with low-risk SAB a switch from intravenous to oral antimicrobial therapy (oral switch therapy, OST) is non-inferior to a conventional course of intravenous therapy (intravenous standard therapy, IST).

**Methods/Design:**

The trial is designed as randomized, parallel-group, observer-blinded, clinical non-inferiority trial. The primary endpoint is the occurrence of a SAB-related complication (relapsing SAB, deep-seated infection, and attributable mortality) within 90 days. Secondary endpoints are the length of hospital stay; 14-day, 30-day, and 90-day mortality; and complications of intravenous therapy. Patients with SAB who have received 5 to 7 full days of adequate intravenous antimicrobial therapy are eligible. Main exclusion criteria are polymicrobial bloodstream infection, signs and symptoms of complicated SAB (deep-seated infection, hematogenous dissemination, septic shock, and prolonged bacteremia), the presence of a non-removable foreign body, and severe comorbidity. Patients will receive either OST or IST with a protocol-approved antimicrobial and are followed up for 90 days. Four hundred thirty patients will be randomized 1:1 in two study arms. Efficacy regarding incidence of SAB-related complications is tested sequentially with a non-inferiority margin of 10 and 5 percentage points.

**Discussion:**

The SABATO trial assesses whether early oral switch therapy is safe and effective for patients with low-risk SAB. Regardless of the result, this pragmatic trial will strongly influence the standard of care in SAB.

**Trial registration:**

ClinicalTrials.gov NCT01792804 registered 13 February 2013; German Clinical trials register DRKS00004741 registered 4 October 2013, EudraCT 2013-000577-77. First patient randomized on 20 December 2013.

**Electronic supplementary material:**

The online version of this article (doi:10.1186/s13063-015-0973-x) contains supplementary material, which is available to authorized users.

## Background

Increasing resistance to antimicrobial agents is recognized as a major health problem worldwide and is compounded by the dearth of new antimicrobial agents currently in development [1]. This threat underscores the need to maximize the clinical utility of existing antimicrobials through more rational prescription, for example, by optimizing the duration of treatment. The SABATO trial will assess whether the duration of intravenous therapy can be reduced in *Staphylococcus aureus* bloodstream infection (SAB).

SAB is a major cause of prolonged antimicrobial therapy. With an approximate incidence of 25 cases per 100,000 population per year, there are about 200,000 cases annually in Europe [2]. Recent data for Western Europe demonstrate a crude mortality of 20-30 % (in-hospital or 30-day mortality) in patients with SAB [2].

In many cases SAB can be cured by antimicrobial therapy. However, SAB differs from other bloodstream infections with respect to SAB-related complications. Relapse, local extension and distant metastatic foci are relatively common events and occur in about 2 to 25 % of infections [3–5]. It is believed that these complications can be minimized by an adequate length of antimicrobial therapy. Therefore, standard treatment schedules are much longer than for other bloodstream infections. For example, a course of at least 14 days of intravenous antimicrobials is considered standard therapy in “uncomplicated SAB” [6–8], whereas even longer courses are required in “complicated” disease. Shorter courses of intravenous treatment are currently not recommended due to the lack of sound clinical evidence. However, these recommendations are based on expert opinion and a few observational studies.

The hypothesis of the SABATO trial is that a switch from intravenous to oral antimicrobial therapy is non-inferior to standard intravenous therapy in patients with low-risk SAB. Therefore, the primary objective of the trial is to demonstrate that oral switch therapy (OST) is as safe and effective as intravenous standard therapy (IST). This will be achieved by comparing the rate of SAB-related complications (relapsing SAB, deep-seated infection with *S. aureus*, or mortality attributable to SAB) within 90 days.

Abbreviated treatment courses or early intravenous to oral switch treatment strategies have been successfully applied to other infectious diseases such as nosocomial pneumonia [9], meningococcal disease [10], and febrile neutropenia [11]. These strategies allow shorter intravenous antimicrobial therapy and offer options for early discharge from hospital. This, in turn, increases the patients’ quality of life, decreases treatment costs, reduces the risk of nosocomial infections and may help to diminish antimicrobial resistance development and spread.

Regarding SAB, there is one controlled randomized study where 16 of 36 patients received short-course therapy consisting of 2 weeks intravenous nafcillin [12]. A difference in complication rate between patients who received 2 or 4 weeks of intravenous antimicrobial treatment could not be detected. However, a meta-analysis of this controlled trial and 10 uncontrolled, epidemiological studies [13] showed potential bias and considerable imprecision and recommended further trials. The effectiveness of oral antimicrobial therapy in SAB has been assessed in a single randomized controlled trial [14]: 104 patients with SAB either received oral fleroxacin plus rifampicin or intravenous study therapy. The cure rate in both groups was similar (82 % versus 80 %), and patients on oral medication were discharged earlier, although they had more drug-related adverse events. This study suggests that orally administered antimicrobials may be as effective as intravenous therapy.

Although not supported by current recommendations, shorter duration of intravenous therapy has become management practice for SAB in some countries: Thwaites et al. reviewed management practices of patients with SAB in eight UK centers and found that 25 % of patients received oral antimicrobials alone for more than 50 % of the treatment duration whereas 16 % of patients received less than the recommended 14 days of therapy [15]. In this study, the efficacy and safety of oral therapy were not assessed.

In this trial, a population of patients with a very low risk of SAB-related complications is selected by rigorous inclusion and exclusion criteria. The selection criteria are based on data from two prospective cohort studies. Data from the INSTINCT (Invasive *Staphylococcus aureus* Infection Cohort) study [16, 17] show a low incidence of SAB-related complications in low-risk patients (3 %; four of 135 patients). A pilot study for the SABATO trial with 236 SAB patients from 10 German study centers [18] provided further evidence for a very low risk of complications, with a single SAB-related complication occurring in 89 patients.

In addition, an early switch to oral medication may also improve patients’ well-being: an abbreviated hospital stay can increase quality of life and reduces the risk of intravascular line-associated inflammation or infection. There may also be benefits for healthcare institutions, such as cost-savings afforded by reduced length of stay or reduced use of outpatient parenteral antimicrobial therapy (OPAT) services.

A successfully performed trial will have a great impact on clinical decision making worldwide. Regardless of its outcome, it will provide a rationale for optimizing treatment of patients with low-risk SAB - a common clinical scenario - that will be integrated into evidence-based treatment guidelines. We will therefore perform a multicenter, center stratified, observer-blinded, randomized, parallel-group, clinical non-inferiority trial with the aim to provide a definitive answer.

## Methods/Design

### Study hypothesis and aims

The aim of the trial is to demonstrate that in patients with low-risk SAB a switch from intravenous to oral antimicrobial therapy is non-inferior to a conventional 14-day course of intravenous therapy. This will be achieved by comparing the rate of SAB-related complications (relapsing SAB, deep-seated infection with *S. aureus*, or mortality attributable to SAB) within 90 days between OST and IST. The secondary objective is to estimate the potential benefit for the patient by evaluating the length of hospital stay after the first positive blood culture, all-cause mortality, and complications of intravenous therapy.

### Setting

The study will be performed in the following 19 university or teaching hospitals in four European countries including the following: in Germany - Uniklinik Köln, University Hospital Freiburg, Vivantes Auguste-Viktoria-Klinikum Berlin, Hannover Medical School, Jena University Hospital, Universitätsklinikum Aachen, Helios Klinikum Krefeld, University of Schleswig-Holstein Lübeck, Klinikum Leverkusen, University Hospital Regensburg, and J.W. Goethe University Hospital Frankfurt; in The Netherlands - University Medical Center Groningen, Amphia Hospital Breda, Sint Elisabeth Hospital Tilburg, Academic Medical Center Amsterdam, and University Medical Center Utrecht; in Spain - Hospital Clínic of Barcelona, Hospital Universitario Virgen Macarena Sevilla, and Hospital Universitario Virgen del Rocío Sevilla; and in the United Kingdom - Nottingham University Hospitals NHS Trust.

### Ethical and regulatory considerations

The trial will be conducted in accordance with the published principles of the guidelines for Good Clinical Practice (ICH-GCP) and applicable legislation (especially the Federal Drug Law (AMG) and the GCP-V). The study protocol was approved by the Ethics Committees of all participating centers and the respective competent authorities (a list is provided in an additional file [see Additional file 1]). Informed consent is obtained from each participant.

The final trial report will follow the CONSORT statement and its extension to non-inferiority and equivalence trials [19].

### Patient selection and sex distribution

**Fig. 1:**
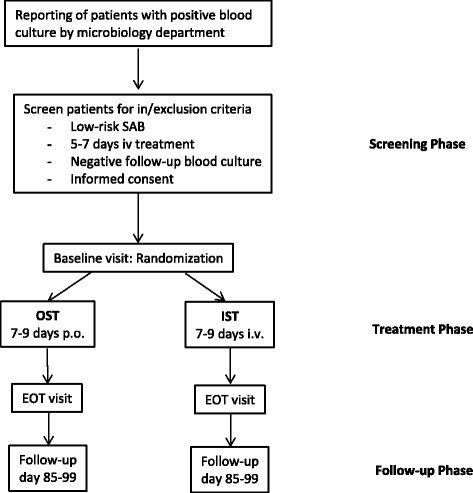
Trial flowchart

Patients with SAB from all disciplines are reported from the microbiological department to the site investigator as soon as *S. aureus* is identified in the blood culture (Fig. 1). Then, individual patients with SAB are assessed for possible enrollment. They will be considered for enrollment when all inclusion criteria and none of the exclusion criteria are fulfilled. All eligible patients are hospital inpatients.

SAB is more frequent in male patients (m:f = 2:1) [20]. The reason for this phenomenon is not known. Since most infections arise from the skin and nasal flora of the patient, this may reflect a higher nasal colonization rate in men [21, 22]. However, mortality does not vary between the male and female sex [20]. Sex-specific differences in efficacy and safety of the study drugs are not expected; however, this will be investigated.

### Inclusion and exclusion criteria

Inclusion and exclusion criteria are designed to select a group of patients with SAB that have a low risk for SAB-related complications. Patients entering the study will have already received 5 to 7 days of adequate intravenous antimicrobial therapy and have no signs and symptoms of complicated *S. aureus* infection prior to enrollment. Patients with a higher *a priori* risk for SAB-related complications are excluded (for example, severe immunosuppression, end-stage renal disease, and presence of non-removable foreign body). Details of inclusion and exclusion criteria are presented in Table 1.

**Table 1:** Inclusion and exclusion criteria

Inclusion criteria	Exclusion criteria
• Age at least 18 years	• Polymicrobial bloodstream infection, defined as isolation of pathogens other than *S. aureus* from a blood culture obtained in the time from two days prior to the first positive blood culture with *S. aureus* until randomization. Common skin contaminants (coagulase-negative staphylococci, diphtheroids, *Bacillus* spp., and *Propionibacterium* spp.) detected in one of several blood cultures will not be considered to represent polymicrobial infection
• Not legally incapacitated	
• Written informed consent from the trial subject has been obtained	
• Blood culture positive for *Staphylococcus aureus* not considered to represent contamination	
• At least one negative follow-up blood culture obtained within 48 to 72 hours after the start of adequate antimicrobial therapy to rule out persistent bacteremia	
	• Recent history (within 3 months) of prior S. aureus bloodstream infection
• Five to 7 full days of appropriate i.v. antimicrobial therapy administered prior to randomization documented in the patient chart. Appropriate therapy has all of the following characteristics:	
	• In vitro resistance of *S. aureus* to all oral or all i.v. study drugs
	• Contraindications in reference document for all oral or all i.v. study drugs
- Antimicrobial therapy has to be initiated within 72 h after the first positive blood culture was drawn.	
	• Previously planned treatment with active drug against *S. aureus* during intervention phase (for example, cotrimoxazole prophylaxis)
- Provided in-vitro susceptibility and adequate dosing (as judged by the principal investigator) preferred agents for pre-randomization antimicrobial therapy are: flucloxacillin, cloxacillin, vancomycin, and daptomycin. However, the following parenteral antimicrobials are allowed: MSSA: penicillinase-resistant penicillins (for example, flucloxacillin and cloxacillin), β-lactam plus β-lactamase-inhibitors (for example, ampicillin + sulbactam, piperacillin + tazobactam), cephalosporins (except ceftazidime), carbapenems, clindamycin, fluoroquinolones, trimethoprim-sulfamethoxazole, doxycycline, tigecycline, vancomycin, teicoplanin, linezolid, daptomycin, ceftaroline, and macrolides. MRSA: vancomycin, teicoplanin, fluoroquinolones, clindamycin, trimethoprim-sulfamethoxazole, doxycycline, tigecycline, linezolid, daptomycin, macrolides, and ceftaroline	
	• Signs and symptoms of complicated SAB as judged by an ID physician. Complicated infection is defined as at least one of the following:
	- deep-seated focus: for example, endocarditis, pneumonia, undrained abscess, empyema, and osteomyelitis
	- septic shock, as defined by the AACP criteria [32], within 4 days before randomization
	- prolonged bacteremia: positive follow-up blood culture more than 72 h after the start of adequate antimicrobial therapy
	- body temperature > 38 °C on 2 separate days within 48 h before randomization
	• Presence of a non-removable foreign body (if not removed two days or more before randomization):
	- prosthetic joint
	- prosthetic heart valve
	- vascular graft
	- pacemaker
	- automated implantable cardioverter-defibrillator
	- ventriculo-atrial shunt
	• Failure to remove any intravascular catheter, which is present when first positive blood culture was drawn within 4 days of the first positive blood culture
	• Severe liver disease
	• End-stage renal disease
	• Severe immunodeficiency:
	- primary immunodeficiency disorders
	- neutropenia (<500 neutrophils/μl) at randomization or neutropenia expected during intervention phase due to immunosuppressive treatment
	- uncontrolled disease in HIV-positive patients
	- high-dose steroid therapy (>1 mg/kg prednisone or equivalent doses given for > 4 weeks or planned during intervention)
	- immunosuppressive combination therapy with two or more drugs with different mode of action
	- hematopoietic stem cell transplantation within the past 6 months or planned during treatment period
	- solid organ transplant
	- treatment with biological
	• life expectancy < 3 months
	• Inability to take oral drugs
	• Injection drug user
	• Expected low compliance with drug regimen
	• Participation in other interventional trials within the previous three months or ongoing
	• Pregnant women and nursing mothers
	• For premenopausal women: Failure to use highly-effective contraceptive methods for 1 month after receiving study drug.
	• Persons with any kind of dependency on the investigator or employed by the sponsor or investigator
	• Persons held in an institution by legal or official order

### Patient randomization

Patients are randomly allocated to treatment arms (1:1) no earlier than 1 day before starting the study drug. This is achieved by a central 24/7 Internet randomization service TENALEA (stratified by study center and with permuted blocks of varying length). Authorized local study staff may login to a secure website, randomize a patient and receive an email with an attached pdf document giving all the details on the allocated treatment. The randomization service is set up and maintained by the Institute of Medical Statistics, Informatics and Epidemiology (IMSIE), University of Cologne.

### Treatment of patients

Treatment of patients in this observer-blinded study follows a pragmatic approach. All study medication is commercially available and approved by the respective national authorities. Study medication is used as marketed in standard dosing regimens. According to the treatment arm, patients will either receive an oral or intravenous study drug. The study drug will be selected by the site investigator from a list of drugs (Table 2), depending on the susceptibility of the respective isolate, expected drug interactions, contraindications, and expected side effects. Investigators will first assess whether the “first-choice” regimen can be given and then consider the alternative regimen. The study drug can also be switched during therapy from first choice to the respective alternative medication. Early discontinuation of study drug or combination therapy (for example, addition of gentamicin, rifampicin, or fosfomycin) is regarded as a protocol violation. Dose adjustments in the individual patients will be performed as judged appropriate by the site investigator following the guidelines in Table 2. Study drug will be discontinued after 7 to 9 days (depending on the length of antimicrobial therapy before randomization) so that patients receive an overall course of 14 days of antimicrobial therapy.

**Table 2:** Choice of study drugs and suggested dosing

OST	Minimum daily dose	Suggested regimen	Acceptable dosing	Dose adjustment
First choice for MSSA and MRSA: trimethoprim-sulfamethoxazole	320/1600 mg	160/800 mg twice a day		Severe renal impairment
Alternative for MSSA: clindamycin	1800 mg	600 mg three times a day		No
Alternative for MRSA: linezolid	1200 mg	600 mg twice a day		No
IST				
First choice for MSSA: flucloxacillin	6 g (in at least four doses a day, or continuous infusion)	2 g four times a day	4 g three times a day	Severe renal impairment
First choice MSSA in Spain: cloxacillin	6 g (in at least four doses a day, or continuous infusion)	2 g four times a day	2 g six times a day	No
Alternative for MSSA: cefazolin	1 g three times a day	2 g three times a day	3 g four times a day	Renal impairment
Alternative for MSSA and first choice for MRSA: vancomycin	as determined by therapeutic drug monitoring	1 g twice a day	20 mg/kg three times a day; loading dose and continuous infusion are accepted	TDM (suggested level: 10 to 20 μg/ml)
Alternative for MRSA: daptomycin	6 mg/kg once per day	6-10 mg/kg once per day		Renal impairment

### Assessment and follow-up

All patients will be followed for 90 days to evaluate efficacy and safety variables. To assess outcome measures, patients will be visited on the ward by the clinical team while in the hospital according to the visit schedule (Table 3). Discharged patients are followed by a structured telephone interview. All data will be recorded on electronic case report forms. Patients on OST can be discharged before the end of therapy (EOT) according to clinical and psychosocial criteria. Patients on IST can only be discharged when an OPAT service is in operation at the local study site.

**Table 3:** Visit schedule

	Screening	Treatment		EOS
Visit number	0	1	2	3
Day	-5 to -1	1	7 to 11(EOT)	85 to 99
Informed consent	X			
Check in/exclusion criteria	X			
Randomization		X		
Demographic data	X			
Medical history	X			
Charlson score	X			
Pitt bacteremia score	X			
Current medication	X	X	X	X
Infective focus	X			
Clinical data				
Physical examination	X	X		
Vital signs	X	X		
Outcome assessment				
SAB-related complications			X	X
Length of stay (in days)			X	X
90-day mortality				X
Safety				
Adverse events		X	X	X
*Clostridium difficile-*associated diarrhea		X	X	X
Complications of iv therapy		X	X	X
Laboratory data				
The following routine laboratory results are documented once from day −3 to 1, if available: Hemoglobin, red and white blood cell count, platelet count, serum sodium, serum potassium, serum creatinine, liver function tests, creatine phosphokinase, C-reactive protein, blood culture	X			
Pregnancy test in premenopausal women	X			

### Outcome measures

The primary endpoint, SAB-related complication, defined as relapsing SAB, deep-seated infection, or attributable mortality, will be derived from patient interviews, laboratory reports, and clinical evaluation. Patients with either condition will be classified as “failure.” SAB-related complications are classified as either “microbiologically documented” or “clinically suspected.” To qualify for a “microbiologically documented” relapsing SAB or deep-seated infection, the *S. aureus* isolate needs to exhibit the same characteristics as the original infecting isolate (based on antimicrobial susceptibility and genotyping tests as appropriate), and not to be considered by the local investigator to represent a contaminant.

Relapsing SAB is defined as positive blood culture for *S. aureus* within the intervention or follow-up period. Proven catheter-related SAB during the follow-up period is not considered relapsing SAB, since catheter-related infection is highly likely the result of a new infection. Catheter-related blood-stream infection is considered “proven,” if (1) the same *S. aureus* strain is recovered from a blood culture and from the catheter tip, or from the blood culture and from pus or a skin swab obtained from the catheter exit site, or if two initial sets of blood cultures positive for *S. aureus* show a positive differential time to positivity and there is no other plausible source of SAB.

Deep-seated infection is any deep-seated focus of *S. aureus* infection resulting from hematogenous dissemination (such as infective endocarditis according to the modified Duke criteria [23], osteomyelitis, or suppurative arthritis). Diagnosis requires either a culture positive with *S. aureus* from the respective site or a blood culture positive with *S. aureus* plus imaging studies showing the presumed focus. Catheter-related infections, superficial skin-and soft tissue or wound infections do not qualify as “deep-seated,” since they are highly likely to result from a new infection.

Death will be attributed to SAB when at least one of the following conditions is present [16]: a positive blood culture for *S. aureus* drawn within 72 h before death, or a persistent focus of deep-seated *S. aureus* infection at time of death, or persistent signs and symptoms of systemic infection at time of death as judged by a study physician, or a post-mortem analysis proving a *S. aureus* related complication as cause of death.

The secondary endpoints are the length of hospital stay (reflects the potential benefits for patients who have been switched to oral medication), 14-day survival, 30-day survival, 90-day survival, and complications related to i.v. therapy, such as chemical or septic thrombophlebitis. The safety of study drugs is assessed by monitoring *Clostridium difficile*-associated diarrhea, and all (severe) adverse events that meet grade 3 or above of the Common Terminology Criteria of Adverse Events (Version 4.0) [24].

### Sample size

Since a non-inferiority margin of 10 percentage points seems to be too high for the clinical question to be addressed, the study sample size was calculated to ensure sufficient power (that is, 80 %) even with a reduced margin of 5 percentage points (as a compromise of precision and feasibility). Both margins, that is, 10 % and 5 %, are tested hierarchically to ensure both sufficient power and type I error control [25].

A large prospective cohort study of 324 SAB patients indicates that patients with a removable focus of infection have a low risk (2.4 %) for late complications [26]. In a similar prospective study of 211 SAB patients with a removable focus of infection, the relapse rate with antimicrobial therapy for less than 14 days (n = 134) was 3.7 % [27]. A meta-analysis of studies performed between 1967 and 1993 reported a combined rate of late complications of 6.1 % in patients with catheter-related infections [13]. When using inclusion and exclusion criteria for low-risk patients, our own studies showed complication rates of 1 % (preSABATO) and 3 % (INSTINCT). Therefore, we estimate an incidence of 2.5 % of late complications in study patients.

Thus, the necessary sample size for each study arm is 165.8 (non-inferiority margin 5 %, one-sided α = 0.05, β = 0.2, one interim analysis at information fraction 0.5 using the O’Brien-Fleming bound 2.373; calculated using ADDPLAN 6.0.1, ADDPLAN GmbH, Cologne). An allowance of 10 % for deaths unrelated to SAB, of 10 % for protocol violations and of 5 % for stratification yields 331.6/0.9/0.9/0.95, approximately 430 patients in total need to be randomized. Of note, if non-attributable mortality (that is, about 10 % within 90 days) were added to the composite endpoint, the sample size needed would approximately double (that is, from 430 to 823 patients). Unfortunately, this cannot be achieved by the clinical network.

At the interim analysis, observations of 2.5 % late complications, that is, 0.025*166/2 ≈ 2 in each group, yields a 98.235 % confidence interval (corresponding to the O’Brien-Fleming bound 2.373) for the difference of -8.2 % to +8.2 % (Newcombe’s method; [28]). At the final analysis (as planned) observations of 2.5 % late complications, that is, 0.025*166 ≈ 4 in each group, yields a 91.765 % confidence interval (corresponding to the O’Brien-Fleming bound 1.678) for the difference of -3.4 % to +3.4 %.

### Statistical analysis

**Fig. 2:**
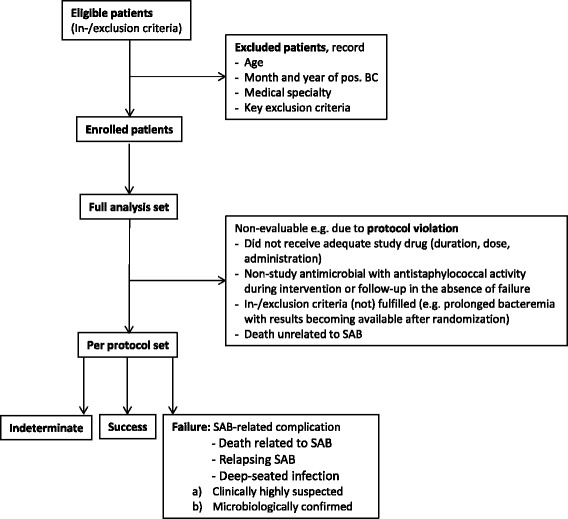
Analysis sets

All analyses will be done on three study populations (Fig. 2). The primary analysis set is derived from the per-protocol (PP) population. This dataset includes all study subjects who were essentially treated according to protocol and reached a defined endpoint in the trial (SAB-unrelated deaths will be excluded). The evaluability of study subjects will be assessed in a blinded manner by the Clinical Review Committee.

The secondary analysis set is derived from the intention-to-treat (ITT) population. This dataset includes all randomized study subjects, analyzed as assigned, with indeterminate and missing outcomes counted as failures. Following current recommendations, CPMP/EWP/558/95 rev 2 [29] and CPMP/EWP/482/99 [30], the primary analysis is based on the per-protocol set; the analysis of the full analysis set (intention-to-treat, all randomized patients) will be of equal importance and should lead to similar conclusions for a robust interpretation.

The tertiary analysis set is the safety population. This dataset includes all study subjects who received any study drug.

The possibly adaptive interim analysis based on 215 included patients (at information fraction 0.5) serves to assess the risk-benefit ratio and may lead to (1) stopping (albeit non-mandatory) due to overwhelming non-inferiority, (2) stopping for futility/safety, (3) continuation as planned, or (4) recalculation of the sample size based on conditional power. Any adaptation of the study design will be based on the observed proportions of SAB-related late complications applying the inverse normal method [31].

### Trial management

The trial will be managed by the clinical project manager, the team of the CTCC, the principal coordinating investigator, and the principal investigators at each site. A Steering Committee was involved in protocol development and will oversee study progress. A Scientific Advisory Committee gives advice on all aspects of the trial, including trial design. A Clinical Review Committee will be responsible for evaluating cases regarding protocol violations, and treatment failures blinded for treatment arm. A Data Monitoring Committee made up of independent experts who are not involved in the conduct of the trial will oversee the safety of the trial subjects in the clinical trial by periodically assessing the safety and efficacy of the trial therapy and will monitor the integrity and validity of the data collected and the conduct of the clinical trial.

### Funding

The clinical trial is funded by the Deutsche Forschungsgemeinschaft (DFG; German Research Foundation; grant number KA 3104/2-1 to A.J.K.). The protocol was endorsed by the European Clinical Research Infrastructure Network (ECRIN).

## Discussion

The SABATO trial will assess whether an early switch to oral medication is as safe and effective as intravenous standard therapy. The trial population consists of patients with a very low risk of SAB-related complications. Many physicians would feel comfortable with a switch to oral medication but current guidelines recommend a full 14-day course of intravenous treatment.

The research question will be addressed in a pragmatic way: patients that have already received 5 to 7 days of adequate intravenous antimicrobial therapy can be enrolled to receive 9 to 7 days of study medication. The route of administration is determined by randomization, but the local investigator decides which study drug to select based on a list of commercially available antimicrobials. Patients that receive OST can be discharged from the hospital.

The primary endpoint-measure - SAB-related complication - reflects the failure rate of antimicrobial therapy in preventing late complications. This includes both relapsing SAB and deep-seated *S. aureus* infection within 90 days and is thus the most appropriate clinical outcome measure.

Death unrelated to SAB was not included in the primary endpoint because this would compromise the power of the trial by variance inflation. However, all causes of mortality will be carefully assessed and compared.

As an alternative endpoint, we considered “microbiological success,” demonstrated by a negative blood culture as a test of cure at EOT. Since patients in this trial have already been treated for 7 days with antimicrobials before randomization, almost all blood cultures obtained at EOT are expected to yield a negative result. Therefore, microbiological success has not been chosen as an endpoint.

Some researchers feel that the trial is not ambitious enough by only changing therapy moderately, and believe that a more aggressive approach, such as testing 7 days of oral medication versus 14 days of intravenous therapy, should have been undertaken. Although, we agree that this is an interesting approach, evidence for its effectiveness is sparse and patient safety is the prime concern in this trial.

Regarding antimicrobial therapy of SAB, many questions are unanswered. Although, early oral switch therapy is often applied in other infections, it has never been assessed in SAB. Therefore, this trial will close gaps in our understanding and regardless of the result the trial will influence medical practice.

## Trial status

The first patient was recruited on 20 December 2013. The last patient is expected to be recruited in December 2016. The University of Cologne provides central trial management and coordination.

## Additional file

Additional file 1:
**List of Ethics Committees and Competent Authorities that approved the trial.** (PDF 190 kb)

## Abbreviations

AMG: Arzneimittelgesetz (German Federal Drug law)
CTCC: Clinical Trial Center Cologne
EOS: end of study
EOT: end of treatment
GCP: Good Clinical Practice
ICH: International Conference on Harmonisation
IRB: Institutional Review Board (Ethics Committee)
IST: intravenous standard therapy
ITT: intention to treat
MRSA: methicillin-resistant *Staphylococcus aureus*
MSSA: methicillin-susceptible *Staphylococcus aureus*
OPAT: outpatient parenteral antimicrobial therapy
OST: oral switch therapy
PCI: Principal Coordinating Investigator
PP: per protocol
SAB: *Staphylococcus aureus* bloodstream infection
