# Supplementary material for: Early oral switch therapy in low-risk Staphylococcus aureus bloodstream infection (SABATO): study protocol for a randomized controlled trial
Source: Trials. 2015 Oct 9;16:450. doi: 10.1186/s13063-015-0973-x (PMC4600306; doi:10.1186/s13063-015-0973-x)
Supplement: Additional file 1: — List of Ethics Committees and Competent Authorities that approved the trial. (PDF 190 kb) [file 13063_2015_973_MOESM1_ESM.pdf]

List of Competent Authorities and Ethics Committees that approved the SABATO trial	
**Country**	**Competent Authority:**
Germany	BfArM<br>Bundesinstitut für Arzneimittel und Medizinprodukte<br>Kurt Georg-Kiesinger-Allee 3<br>53175 Bonn<br>Germany
Spain	AEMPS<br>Agencia Espanola de Medicamentos y Productos Sanitarios<br>Calle Campezo 1<br>Edificio 8<br>E-28022 Madrid<br>Espana
The Netherlands	CCMO<br>Centrale Commissie on Mensgebonden Onderzoek<br>Postbus 16302<br>2500 BH Den Haag<br>The Netherlands
**Site**	**Ethics Commission**
Köln<br>(Lead EC)	Geschäftsstelle Ethikkommission<br>Universität zu Köln<br>Gebäude 5<br>Kerpener Str. 62<br>50937 Köln<br>Germany
Aachen	Geschäftsstelle der Ethik Kommission des Universitätsklinikums<br>Pauwelstraße 30<br>352074 Aachen<br>Germany
Berlin	Landesamt für Gesundheit und Soziales Berlin<br>Fehrbelliner Platz 1<br>10707 Berlin<br>Germany
Frankfurt	Geschäftsstelle der Ethikkommission des Fachbereichs Medizin<br>der Johann Wolfgang Goethe-Universität Haus 1<br>Theodor-Stern-Kai 7<br>60590 Frankfurt/Main<br>Germany
Freiburg	Geschäftsstelle der Ethikkommission der Albert-Ludwigs-Universität<br>Freiburg<br>Engelberger Str. 21<br>79106 Freiburg<br>Germany
Jena	Geschäftsstelle der Ethik-Kommission der Friedrich-Schiller-Universität<br>Jena Bachstr. 18<br>07740 Jena<br>Germany

Krefeld	Geschäftsstelle der Ethik Kommission der<br>Ärztekammer Nordrhein<br>Tersteegenstr. 9<br>40474 Düsseldorf<br>Germany
Leverkusen	Geschäftsstelle der Ethik Kommission der<br>Ärztekammer Nordrhein<br>Tersteegenstr. 9<br>40474 Düsseldorf<br>Germany
Lübeck	Geschäftsstelle der Ethik-Kommission der Universität zu Lübeck<br>Ratzeburger Allee 160<br>Haus 21<br>23562 Lübeck<br>Germany
Regensburg	Geschäftsstelle der Ethik-Kommission der Medizinischen Fakultät<br>Universitätsklinikum Regensburg<br>Franz-Josef-Strauß-Allee 11<br>93053 Regensburg<br>Germany
Utrecht<br>(National Lead)	Medisch Ethische Toetsingscommissie<br>UMC Utrecht<br>Huispostnummer D 01.343<br>Postbus 85500<br>3508 GA UTRECHT<br>The Netherlands
Amsterdam	Medisch Ethische Toetsingscommissie<br>AMC Amsterdam<br>The Netherlands<br>Postbus 22660<br>1100 DD Amsterdam
Groningen	Medisch Ethische Toetsingscommissie<br>Postbus 30001<br>9700 RB Groningen<br>The Netherlands
Breda	Medisch Ethische Toetsingscommissie<br>Stichting Amphia<br>Molengracht 21<br>4818 CK Breda<br>The Netherlands
Barcelona	CEIC Hospital Clínic<br>C. Villarroel, 170<br>Sótano. Escalera 6B<br>08036 Barcelona<br>Spain
Sevilla	CEIC de los hospitales Universitario Virgen Macarena-Virgen del Rocío<br>Avda. Manuel Siurot<br>41013 Sevilla<br>Spain
